# Mindful with Your Baby: Feasibility, Acceptability, and Effects of a Mindful Parenting Group Training for Mothers and Their Babies in a Mental Health Context

**DOI:** 10.1007/s12671-017-0699-9

**Published:** 2017-04-13

**Authors:** Eva S. Potharst, Evin Aktar, Marja Rexwinkel, Margo Rigterink, Susan M. Bögels

**Affiliations:** 1 UvA Minds, Academic Outpatient (Child and Adolescent) Treatment Center, University of Amsterdam, Amsterdam, The Netherlands; 2 Research Institute of Child Development and Education, University of Amsterdam, Nieuwe Achtergracht 127, WS 1018 Amsterdam, The Netherlands; 3 Clinical Psychology Unit, Leiden University, Leiden, The Netherlands; 4 Infant Mental Health Center OuderKindLijn, Amsterdam, The Netherlands; 5 Medical Pedagogical Center ‘t Kabouterhuis, Amsterdam, The Netherlands; 6 Research Priority Area Yield, University of Amsterdam, Amsterdam, The Netherlands

**Keywords:** Mindfulness, Parenting, Infants, Postpartum depression

## Abstract

Many mothers experience difficulties after the birth of a baby. Mindful parenting may have benefits for mothers and babies, because it can help mothers regulate stress, and be more attentive towards themselves and their babies, which may have positive effects on their responsivity. This study examined the effectiveness of *Mindful with your baby*, an 8-week mindful parenting group training for mothers with their babies. The presence of the babies provides on-the-spot practicing opportunities and facilitates generalization of what is learned. Forty-four mothers with their babies (0–18 months), who were referred to a mental health clinic because of elevated stress or mental health problems of the mother, infant (regulation) problems, or mother-infant interaction problems, participated in 10 groups, each comprising of three to six mother-baby dyads. Questionnaires were administered at pretest, posttest, 8-week follow-up, and 1-year follow-up. Dropout rate was 7%. At posttest, 8-week follow-up, and 1-year follow-up, a significant improvement was seen in mindfulness, self-compassion, mindful parenting, (medium to large effects), as well as in well-being, psychopathology, parental confidence, responsivity, and hostility (small to large effects). Parental stress and parental affection only improved at the first and second follow-ups, respectively (small to medium effects), and maternal attention and rejection did not change. The infants improved in their positive affectivity (medium effect) but not in other aspects of their temperament. Mindful with your baby is a promising intervention for mothers with babies who are referred to mental health care because of elevated stress or mental health problems, infant (regulation) problems, or mother-infant interaction problems.

## Introduction

Giving birth to a new baby is a transformational process that brings changes in every aspect of a woman’s life. The transition to motherhood comprises many developmental tasks, including taking responsibility over the child day and night, forming a bond with the baby, adapting to changing relationships with the partner, forming a mother identity, finding and accepting support, finding a balance with other activities, and learning mothering (Nelson 2003). Learning mothering encompasses an endless list of abilities, including regulating the baby’s (emotional) states, and the mother’s own emotional reactions to the demands of the baby. When a new mother perceives that the demands she faces exceed available coping resources, she will experience stress (Lazarus and Folkman 1986), and chronic stress can result in mental health problems (Lupien et al. 2009). These difficulties have a higher occurrence in the presence of risk factors, such as a preterm birth (Nelson 2003), attachment insecurity of the mother (Feeney 2003), or lack of social support (Crnic et al. 1986). Also, difficult infant temperament can be a risk factor for mental health problems in mothers, even in the first month of a baby’s life (Britton 2011).

Although immediately after giving birth there is a rise in life satisfaction, over the many months to follow, this seems to decrease (Luhmann et al. 2012). Approximately half of women experience *maternity blues* in the first couple of weeks post partum, a temporary mood disturbance with accompanying insomnia, fatigue, irritability, sadness, anxiety, and confusion (Reck et al. 2009). Although maternity blues symptoms are usually transitory, postpartum blues are not insignificant, as they constitute a risk factor for anxiety disorders and depression (Reck et al. 2009) and problems in maternal attachment to the infant (Nagata et al. 2000). As many as 19% of women experience depression in the first 3 months after giving birth to a baby (Gavin et al. 2005). Mood problems are not the only risk after giving birth: 9% of women develop a full-blown posttraumatic stress disorder (Alcorn et al. 2010), and an additional 18% have symptoms of posttraumatic stress. Around a quarter of women have other forms of clinically significant anxieties (Alcorn et al. 2010). Obsessive-compulsive disorder and generalized anxiety disorder, in particular, have a heightened prevalence in the postpartum period (Ross et al. 2006). Maternal stress or mental health problems may interfere with the mother’s ability to attune, regulate, and appropriately respond to their infant, which, in turn, increases the risk for problems in emotional, social, and cognitive development of the child (Crnic et al. 1986; Siegel and Hartzell 2003). High maternal stress (Pesonen et al. 2005) and maternal mental health problems (Henrichs et al. 2009; Titotzky et al. 2010) are predictive of infant temperamental difficulties.

The transition to motherhood is not only a period in which the chances of stress and mental problems are elevated; it is also a time with the potential for emotional growth for the mother (Feeney 2003). The importance of timely intervention in the case of vulnerabilities or the emergence of problems after the birth of a baby is unequivocal (Bennett and Indman 2003). This has the potential to improve maternal sensitivity towards her infant and prevent long-term consequences of maternal stress for the child (Bakermans-Kranenburg et al. 2003). At present, a variety of interventions for mothers with babies who experience stress in motherhood are already available. Depending on the nature of the problem, an intervention is chosen with either a primary focus on the mother, on the baby, or on the interaction between mother and baby.

In the case of mental health problems of the mother, the intervention of choice often focuses on the mother. When a mother has a depression or anxiety disorder, pharmacological treatment is often prescribed (Misri et al. 2004). However, the efficacy of antidepressants in postpartum depression is not unequivocal (Sharma and Sommerdyk 2013), and possible effects of antidepressant drugs in breast milk on the nursing infant cannot be excluded (Gentile et al. 2007). Therefore, effective non-pharmacological treatments may offer a preferable alternative to medication in the postnatal period (Dimidjian and Goodman 2009). Individual psychotherapy for the mother often alleviates the mother’s psychological complaints, but the baby may not be taken along in the process of change. A meta-analysis showed that individual psychotherapy for mothers is not effective in improving mothers’ sensitivity (Kersten-Alvarez et al. 2011). For mothers whose primary worries are focused on infant behavior, for example eating or sleeping, behavioral interventions are available that focus on the problem behavior of the child. However, attention to factors that may prevent improvement (e.g., the mother-child relationship or the inner world of the mother) may not be part of these programs. Another disadvantage of behavioral interventions is that they may not fit with the mother’s ideas about parenting and may undermine the mother’s intuition about what is right for herself and her baby (Douglas and Hill 2013). There are also interventions that focus on the mother-infant relationship or are aimed at improving maternal sensitivity, such as video-home training or parent-child interaction therapy. A disadvantage of these interventions is that mothers may miss concrete tools to deal with stressful situations and accompanying emotions.

An intervention that is designed to cope with stress is the mindfulness-based stress reduction (MBSR) training (Kabat-Zinn 1990). MBSR has shown to have beneficial effects in dozens of randomized controlled trials (De Vibe et al. 2012). Mindfulness can be defined as “paying attention in a particular way: on purpose, in the present moment, and non-judgmentally” (Kabat-Zinn 1994, p. 4). The MBSR training consists of meditations; inquiry, in which participants share about their experiences during meditations; and psychoeducation. This training is applicable and is being used worldwide for many different mental and somatic complaints. Mindfulness-based cognitive therapy (MBCT; Segal et al. 2002, 2012) is an important adaptation of MBSR, developed for people with (recurrent) depression. Dimidjian and Goodman (2009), that have reviewed the evidence base for non-pharmacologic interventions for depression during pregnancy and the postpartum period, stated that the application of MBCT to at-risk perinatal women may significantly enhance prevention efforts. When MBCT is applied in this group of new mothers, adaptations might be beneficial. Mothers should be offered mindfulness not only as a way to relate differently to their own experience but also to their babies. That is, the mindfulness training should be transformed into a mindful parenting training.

In a training in mindful parenting, a term that was introduced by Kabat-Zinn and Kabat-Zinn (1997), parents learn to cultivate mindfulness (thus intentionally bring non-judgmental awareness to their experience in the present moment) in parenting and in the relationship with their child. Bögels and Restifo (2013) adapted the MBSR and MBCT trainings to a mindful parenting training for parents in a mental health care context. This training has been applied to different groups of parents (e.g., Bögels et al. 2014; Meppelink et al. 2016), but no adaptations so far addressed mothers who experience stress in taking care of their babies in particular.

Another adaptation to MBCT and MBSR was made to develop the Mindful Motherhood intervention for pregnant women; in a small randomized controlled trial, this intervention was shown to be effective in reducing anxiety and negative affect during pregnancy (Vieten and Astin 2008). Qualitative research showed that mothers that participated in the Mindful Motherhood intervention during their pregnancy went on to use mindful awareness in their relationships with their babies (Krongold 2011). Participants reported that mindful awareness helped them to reflect upon their experiences, to cope with distress, and to enhance pleasure with their babies.

Yet another mindfulness-based intervention (an adaptation to MBSR) for pregnant women is the Mindfulness-Based Childbirth and Parenting (MBCP) program. Two pilot studies among pregnant women showed that anxiety and depression symptoms decreased and that mindfulness increased after participating in MBCP (Duncan and Bardacke 2010; Dunn et al. 2012). Improvements maintained at follow-up 6 weeks post partum (Dunn et al. 2012). Qualitative reports from participants also showed perceived benefits of mindfulness in early parenting (Duncan and Bardacke 2010; Dunn et al. 2012). Another qualitative study showed that participants reported that they still practiced mindfulness 3 years after the program and that mindfulness practice improved their self-regulation and attunement to their child (Shaddix 2014).

Aforementioned follow-up measurements in the postnatal period of studies evaluating the Mindful Motherhood intervention and MBCP program show that mindfulness practice might be useful for mothers with babies. However, more rigorous changes to the program might be needed when mindfulness is taught to mothers in the postnatal period, as not only the mother’s needs but also the baby’s are at stake. Hassan (2014) teaches mindfulness to mothers with infants in Mindful Mothers’ Groups; however, to the extent of our knowledge, no research on these groups is yet available. Also, Reynolds (2003) has been facilitating mindful parenting groups, in which parents learn to quietly observe their babies with curiosity and to reflect on what they notice both in the babies and in themselves during the observation. Reynolds (2003) offered mindful watching to the participating parents, to facilitate self-regulation of, and co-regulation between parent and baby, and improve parents’ mentalizing capacity. For this intervention, which is rooted in the infant mental health (IMH) and psychoanalytical tradition, only anecdotal evidence is available, which seems to point to a positive impact on the parent-child relationship. Although the groups aim at enhancing mindful awareness in parents, mindfulness theory and meditations are not explicitly taught.

A manualized mindfulness training that is adjusted to the needs of both mothers and babies might be of added value for women who experience stress (whether it is because of their own mental health problems, infant (regulation) problems, or mother-infant interaction problem) in mothering their baby. It may teach them tools that they can use to deal with stressful emotions and be more attentive and responsive to their own needs and the needs of their babies. Furthermore, it may offer mothers a holding environment in which they can safely reflect not only on behavioral aspects of their relationship to their babies but also on the inner world of both themselves and their babies. It may support the mothers’ intuition because parenting behaviors are not prescribed and no standpoint on different parenting methods that mothers may choose to employ is taken.

Although the literature about the effects of mindfulness training on mothers with babies is scarce, there is some scientific support for the benefits that mindfulness might have for mothers and babies. Maternal mindfulness during pregnancy has not only shown to be associated with less maternal prenatal and postnatal emotional distress but also with better social-emotional development of their babies (Braeken et al. 2016; Van den Heuvel et al. 2015a), less difficult infant temperament, and improved infant neurodevelopmental outcomes (Van den Heuvel et al. 2015a, b). Also, postnatal mindfulness in parenting (not mindfulness in general) has shown to be predictive of infant stress regulation. In families with high life stress, maternal mindful parenting assessed 3 months post partum was associated with lower infant cortisol at 6 months (Laurent et al. 2016).

Siegel and Hartzell (2003) used insights from the research fields of both attachment and neurobiology to explain how mindfulness might help parents to communicate well, and form secure relationships with their children, and how this impacts different parts of the child’s developing brain. When parents are preoccupied with the past or worried about the future, they are not available for their child to connect with them. Practicing mindfulness means practicing focusing attention on what is happening in the present moment, awareness of the inner experience, being open to the inner experience of the child, and recognizing the separateness of the child’s experience to one’s own experience. Self-attunement, self-care, and self-compassion of the parent form the basis for connecting with, and compassion for others, including a (young) child (Siegel 2007; Siegel and Hartzell 2003). When parents are mindful, they are able to direct their behavior, taking into consideration the (emotional) well-being of the child, and when parents communicate mindfully, they open the space for a child to gain a sense of self, learn to trust others, and build relationships (Siegel and Hartzell 2003).

Cree (2010) explained how improving compassion in compassion focused therapy, an intervention that is related to mindfulness, can improve mother-infant attachment. Starting points are the three major affect regulation systems that interact with each other: a threat-based system and two positive systems, namely an incentive-seeking system and a soothing system. When the threat-based system is highly activated for a long period, the soothing system is suppressed. Cree (2010) described how the soothing system can be stimulated, which will stimulate oxytocin production. Oxytocin then inhibits the threat-based system and opens the door to bonding of the mother to the infant and the development of a secure attachment and relationship between them.

The goal of the current study is to evaluate the effects of a mindful parenting group training, *Mindful with your baby*, for mothers and babies who were referred to a mental health clinic because of elevated stress or mental health problems of the mother, (regulation) problems of the baby, or mother-infant interaction problems. The Mindful with your baby training makes use of the same general meditation exercises and similar attitudinal foundations as the regular mindful parenting, MBCT, and MBSR trainings but is adapted to the presence of the babies and the themes that play a role for most mothers with a baby. We used a longitudinal design, with a pretest, posttest, 8-week follow-up, and 1-year follow-up. We hypothesized that Mindful with your baby would be feasible, acceptable, and effective in improving maternal mindfulness, mindful parenting, self-compassion, well-being, psychopathology, parenting stress, lack of confidence, warm and negative behavior towards the infant, and infant temperamental behavior. We expected that these effects would be maintained up to 8 weeks and 1 year after the training had ended.

## Method

### Participants

Forty-four mothers (*M*_age_ = 33.6 years; *SD* = 4.6) with 0- to 18-month-old infants (*M*_age_ = 10.3 months; *SD* = 4.6; 22 boys (50%) and 22 girls; 28 firstborns (64%)) were referred to Mindful with your baby because of maternal mental health problems or stress related to motherhood. Most mothers and babies lived with the father of the baby (37; 84%), while other mothers lived alone with their baby (5, 11%), lived with the baby and the grandparents (1; 2%), or in assisted living (1; 2%). Their ethnicity was Dutch for 29 (66%), European for 4 (9%), and non-European for 11 (25%) of the mothers. Concerning the level of education, 7 (16%) mothers had a master’s degree, 22 (50%) a bachelor’s degree, 8 (18%) an associate degree, 6 (14%) high school, and 1 (2%) primary school. Fourteen mothers (32%) were working at a job at the time of the training, 20 (45%) were on sick leave, 7 (16%) were stay-at-home mothers, and 3 (4%) were on maternity leave. The majority of mothers had had psychological or pedagogic support (often IMH treatment) prior to Mindful with your baby (and after the birth of their baby) (38; 86%). Twenty-seven mothers (61%) received at least two sessions of other forms of psychological or pedagogic support (often IMH treatment) during the training or in the follow-up period. Our clinical impression was that both forms of mental health care seemed to facilitate the other.

Mindful with your baby was given in primary (two groups) or secondary (eight groups) mental health care centers. The starting dates of the trainings were between May 2013 and September 2016. Thirty-seven (84%) of the mothers had a mental health disorder, such as a depression (19; 43%) or an anxiety disorder (13; 30%). Diagnoses of the mothers were obtained by clinical assessment. Twenty-four (55%) of the babies showed (regulation) problems, such as excessive crying (8; 18%) or sleeping problems (12; 27%). Twenty-four (55%) of the mothers had experienced elevated stress related to pregnancy or birth (such as medical complications during birth (7; 16%) or previous unresolved miscarriages (4; 9%)), and 36 (82%) of the mothers experienced stress in family relations or circumstances (such as relationship problems with the partner (9; 21%) or financial problems (4; 9%)).

Two mothers participated in the training a second time, because they felt a need for extra support in their process. One of these mothers felt that she profited a lot from the training for her own stress complaints and asked for another training to work on improving the relationship with her baby son. Only data from the first training were used from this mother. The second mother participated for a second time because of a sudden severe illness of her husband during the first training which caused a lot of extra stress. This mother filled in only the pretest for the first training, while she completed three measurement occasions for the second training. The data of the second training were used from this mother.

### Procedure

#### Assessments

After obtaining informed consent, the first assessment took place in the week before the start of the training. The second, third, and fourth assessments were administered in the week after the end of the training, at the time of the follow-up session 8 weeks after the end of the training, and 1 year after the training, respectively. All training participants agreed to participate in the research; four (9%) of them however filled in none of the questionnaires. Another three participants (7%) did not finish the training and did not complete the posttest and follow-up measurements; these participants were excluded from the analyses. Therefore, of the 44 training participants, 37 were also research participants. The participation rate of the research participants was 97% at pretest, 97% at posttest, and 84% at follow-up. The 1-year follow-up had not yet been administered to the last two groups. Of the 28 research participants that had been administered the 1-year follow-up, the participation rate was 64%. The exact number of questionnaires that were completed per measurement occasion is displayed in Table 2. Questionnaires were completed at home online by the participating mother (duration approximately 45 to 60 min per assessment moment).

#### Training

The Mindful with your baby program is an adaptation for mothers with a baby of the mindful parenting training (Bögels et al. 2014), which is based on MBSR (Kabat-Zinn 1990) and MBCT (Segal et al. 2002, 2012). Mindful with your baby is adapted to the presence of the babies and the themes that play a role for most mothers with a baby. An example of an adjusted theme is that of *closeness and distance* which replaces the theme of rupture and repair in the regular mindful parenting training (Bögels et al. 2014). An example of an adjusted meditation exercise is a mindful seeing exercise with attention for the baby, in which the mothers learn to (1) focus friendly and curious attention on the baby, notice distractions, and bring back friendly their attention to the baby; (2) notice their own inner reaction; and (3) take the perspective of the baby. An example of an adjusted reading handout that is part of the home practice is a text about how to use mindfulness when the baby cries.

The training Mindful with your baby consists of eight weekly 2-h sessions, plus a follow-up session 8 weeks later. The first and the fifth sessions are with the mothers only in order to have enough time and attention to get acquainted with and deepen the experience and teachings of mindfulness and the contact with the group. The rest of the sessions are with both mothers and babies present. By having the babies present during the majority of the training, the course becomes an *on-the-job training*. As the mothers wish to use mindfulness in contact with the baby, the presence of the babies facilitates generalization of what has been learned. Mothers can practice becoming aware of their own experience while the babies are present, as well as focusing a friendly, open attention on the baby and the signals that he shows, and they can practice with applying mindfulness in stressful situations, which arise spontaneously when bringing the babies into the room.

Groups were led by a mindfulness trainer (EP for the majority of groups), who was responsible for leading the training, and an IMH specialist (MR for the majority of groups), who was responsible for monitoring and keeping in mind the well-being of all mother-baby dyads and the well-being of the babies during the formal meditation in which the mothers close their eyes. Sessions with the babies have a similar composition. First, a formal meditation is done. When the babies are present, the instructions of the formal meditations are adjusted so that it is clear for the mothers that the meditation is not about *shutting out* their babies but merely about being aware of the direction their attention tends to go, keeping in touch with herself while the baby is present and making conscious and flexible decisions about directing their attention, according to the needs of the baby. Halfway into these 10- to 15-min meditations, the mothers open their eyes with full attention to look at their babies and check how they are and whether they need something from them, after which the mothers close their eyes again and notice what their experience of looking and making contact was. The meditation is followed by inquiry and a discussion of the home practices. After that, a 15-min break is taken, with something to drink and eat for mothers and babies. After the break, we introduce the new theme, for example by doing a visualization exercise. Usually, some babies start to get tired near the end of the session; this is a good moment to give full attention to them using a seeing meditation in which they are the focus of the attention. While the mothers watch their baby, the mindfulness trainer gives instructions (for example, to notice whether their attention sticks to one aspect of the baby and then to widen the attention to see the baby as a whole). The theme of the session is also integrated in this exercise (for example, in the session on distance and closeness, mothers are invited to notice fluctuations in feelings of distance and closeness). Experiences are shared in inquiry afterwards. The sessions are finished by explaining the new home practices. When stress arises for mothers during a session, a 3-min breathing space is practiced with the group, which provides mothers with a positive experience and understanding of the use of a 3-min breathing space. Home practice consists of (1) reading handouts about mindfulness and mindful parenting for mothers with a baby, (2) formal meditation to be practiced as much as possible when the baby is asleep or someone else takes care of the baby, (3) informal meditation, and (4) mindful parenting exercises.

### Measures

#### Mindfulness

Five facets of mindfulness were assessed using the short form of the Dutch version of the Five Facet Mindfulness Questionnaire (FFMQ; Baer et al. 2006; De Bruin et al. 2012). Items were scored on a 5-point Likert scale ranging from 1 (never or very rarely true) to 5 (very often or always true). Although the short form comprises of only 24 of the original 39 items, the short form also showed a five-factor structure: observing, describing, acting with awareness, non-judging, and non-reactivity. The psychometric properties of the original scale were good in both a meditating sample and a non-meditating sample (De Bruin et al. 2012). In the current study, Cronbach’s alphas were .88 for the full scale and .77, .83, .80, .62, and .79 for the subscales, respectively.

#### Mindful Parenting

To measure mindful parenting, the Dutch version of the Interpersonal Mindfulness in Parenting Scale (IM-P) was used (De Bruin et al. 2014; Duncan 2007). Of the original 31-item self-report questionnaire, four items (items 4, 7, 8, and 28) were left out that were not applicable for mothers with a baby. The items were scored on a 5-point Likert scale, ranging from 1 (never true) to 5 (always true). The original IM-P consists of five hypothesized subscales that were not factor analytically validated. In a Dutch validation study (De Bruin et al. 2014), however, factor analysis revealed a structure of six dimensions: listening with full attention, compassion for the child, non-judgmental acceptance of parental functioning, emotional non-reactivity in parenting, emotional awareness of the child, and emotional awareness of self. The factor structure of IM-P adjusted for babies is not known. De Bruin et al. (2014) showed satisfactory reliability. Cronbach’s alphas in the current study were .91, .83, .73, .81, .76, .85, and .65 for the total scale and subscales, respectively.

#### Self-Compassion

To measure self-compassion, the 3-item Self-Compassion Scale (SCS-3) was used (Raes and Neff, unpublished manuscript). The three items represent the three different subscales of the Self-Compassion Scale (SCS; Neff 2003): common humanity, overidentification, and self-judgment. The items were scored on a 5-point Likert scale, ranging from 1 (almost never) to 5 (almost always). The internal consistency of this 3-item scale (Cronbach’s alpha) was found to be .74, and the correlation with the total score of the 12-item short form of the SCS was .90 (Raes et al. 2011; Raes and Neff, unpublished manuscript). In the current study, Cronbach’s alpha was .62.

#### Well-Being

Maternal well-being was measured using the Dutch version of the Well-Being Index WHO-5 (Hajos et al. 2013). The WHO-5 consists of five items that are rated on a 6-point Likert scale, ranging from 0 (totally not) to 5 (constantly). Scores are summated and multiplied by 4, to transform them to a 0–100 scale. A score of 50 or below is regarded as a subclinical score (low mood) and a score of 28 or below as a clinical score (depression). A recent systematic review of the literature on the WHO-5 (and translated versions) showed that the WHO-5 has high clinimetric validity and can be used as an outcome measure in studies evaluating interventions (Topp et al. 2015). In the current study, Cronbach’s alpha was .83.

#### Psychopathology

Mothers’ psychopathology was assessed with a Dutch version of the Adult Self-Report (ASR; Achenbach and Rescorla 2003). This self-report scale for adults (18 to 59 years) contains 126 items on problem behaviors, which are rated on a 3-point scale: 0 (not true), 1 (somewhat or sometimes true), and 2 (very true or often true). An example of an item is “I cry a lot.” The items can be scored on eight syndrome scales (withdrawn, somatic complaints, anxious/depressed, rule-breaking behavior, aggressive behavior, intrusive, thought problems, and attention problems), which can be summed up to an internalizing score, an externalizing score, and a total problem score. Those are regarded as subclinical and clinical when *T* scores exceed 59 and 63, respectively. The syndrome scales are regarded as subclinical when *T* scores exceed 64 and clinical when *T* scores exceed 69. Good psychometric properties have been shown for the American version of the ASR. Also, Meppelink et al. (2016) reported excellent internal consistency in a Dutch group of parents (Cronbach’s alpha of .95). In the current study, Cronbach’s alpha of the total scale was .96, and .92 and .87 for the internalizing and externalizing scales, respectively. The subscales had alphas of .83, .81, .88, .80, .81, .71, .66, and .87, respectively.

#### Parenting Stress and Lack of Confidence

Parenting stress was assessed with the Dutch Parenting Stress Index (PSI), based on the American Parenting Stress Index (Abidin 1983; de Brock et al. 1992). We used a combination of the short form of the PSI and seven extra items needed for the 13-item subscale sense of incompetence, measuring the extent to which the parent feels incompetent in parenting the child. Parents rated each item on a 6-point Likert scale, ranging from 1 (totally disagree) to 6 (totally agree). Scores were summated and regarded clinical (very high) above the 95th percentile, and scores above the 85th percentile were regarded as subclinical (high). The Dutch PSI possesses good reliability (de Brock et al. 1992). In the current study, Cronbach’s alphas were .94 for the short form and .92 for subscale sense of incompetence.

#### Maternal Warmth and Negativity Towards the Baby

Maternal warmth and negativity towards the baby was assessed by the scales warmth and negativity of the Comprehensive Parenting Behavior Questionnaire 1-year version (CPBQ-1; Majdandžić et al. 2015). The warmth scale consists of 16 items and assesses the extent to which the parent has positive attention for the baby (subscale attention), shows affection to the baby (subscale affection), and is responsive towards the baby (subscale responsivity). The negativity scale consists of seven items and assesses the extent to which the parent communicates rejection (subscale rejection) or hostility (subscale hostility) towards the baby. Items were rated on a 5-point Likert scale ranging from 1 (totally not applicable) to 5 (completely applicable). In the current study, Cronbach’s alphas were .92 for warmth and .80 for negativity. For the subscales, Cronbach’s alphas were .60 (attention), .96 (affection), .73 (responsiveness), .62 (rejection), and .65 (hostility).

#### Infant Temperament

Infant temperament was assessed using the very short form (Putnam et al. 2014) of the Dutch version of the Infant Behavior Questionnaire-Revised (IBQ-R; Gartstein and Rothbart 2003). Items were scored on a 7-point Likert scale ranging from 1 (never) to 7 (always). The very short form comprises of 37 items of the original 191 and covers three broad components of the IBQ-R: positive affectivity/surgency, orienting/regulatory capacity, and negative emotionality. The IBQ-R has been developed for infants between 3 and 12 months of age and can be used for children up to 18 months. Because of this limited age range, the IBQ-R was not included in the 1-year follow-up. Although the Early Child Behavior Questionnaire (ECBQ; Putnam et al. 2006), which is used to measure the temperament of toddlers aged 1.5 to 3 years, is relatively comparable to the IBQ-R and could have been used at the 1-year follow-up, scores on the IBQ-R and ECBQ cannot be combined in a single dataset (Putman, personal communication, January 6, 2016). The internal consistency of the IBQ-R was acceptable, and interparent agreement was comparable to that obtained with standard IBQ-R scales (Putnam et al. 2006). Cronbach’s alphas in the current study were .87, .71, and .79 for the three components, respectively.

#### Evaluation

At posttest, participants completed a program evaluation, which was an adapted version of the stress reduction program evaluation, developed at the Center for Mindfulness of the University of Massachusetts Medical School, to evaluate how they appreciated Mindful with your baby.

### Data Analyses

Inspection of distribution of differences (scores posttest minus pretest) indicated sufficient normality, skewness, and kurtosis of all variables of <|3.5|, except for IM-P total score and subscale compassion for the child, and ASR total score, externalizing scale, and subscales anxious/depressed, rule-breaking behavior, and aggressive behavior. Of these (sub)scales, one, one, two, two, two, one, and two outliers respectively (>2.5 *SD* or <−2.5 *SD*) were replaced by the next most extreme value at the end of the distribution of the difference scores of these (sub)scales. Hypotheses on the effects of the training on all outcomes were tested with multilevel regression models that are known to accommodate missing data (Bagiella et al. 2000). The structure of the multilevel models for both parent and infant outcomes consisted of the repeated measurements of these outcomes across the measurement points (at pretest, posttest, 8-week follow-up, and 1-year follow-up; fixed effects) nested within the mother-infant dyad. Measurement occasions were dummy coded with pretest scores as reference. Because the group that the mothers and babies participated in may have influenced the effects of the training (as the groups had, for example, different locations, mindfulness trainers, IMH specialists, group composition, and group dynamic), we controlled for the variable group (as both random and fixed effects) in each model. Infant age was also added to the models as a control variable (fixed effect). Of the control variables (group and infant age), only significant effects were retained in the models. The intercept was a fixed effect in all models. Scores on all outcomes were standardized across assessments. Parameter estimates can be interpreted as effect sizes. Effects were regarded as significant when *p* <.05.

## Results

### Feasibility and Acceptability

An acceptable number of participants (3; 7%) did not finish the training. The session attendance rate of the participants that finished the training (*n* = 41) was calculated by dividing the number of attended sessions by the total number of sessions. The average session attendance rates were 90% for the eight weekly sessions, 74% for the follow-up session, and 88% for the combination of the eight weekly sessions and the follow-up. Mindful with your baby appears to be a feasible program. Acceptability was high as well, which was shown by the results of the evaluation, filled in at posttest by 34 (92%) of the research participants (see Table 1).

**Table 1:** Evaluation of the Mindful with your baby training at posttest (*n* = 34)

Question	Yes	No		
Do you feel you got something of lasting value as a result of taking this training?	34 (100%)	0 (0%)		
Have you made any changes in lifestyle or parenting as a result of the training?	29 (85%)	5 (15%)		
Did you become more ‘conscious’ in parenting?	30 (88%)	4 (12%)		
Is it your intention to keep on practicing the formal meditations?	32 (94%)	2 (6%)		
Do you have the intention to keep practicing mindful parenting?	33 (97%)	1 (3%)		
	Never	1–2 times	3–4 times	5–7 times
How often did you practice the formal meditations at home during the training usually?	0 (0%)	6 (18%)	20 (60%)	8 (24%)
Has there been change as a result of the training in	Clear	Some	No	Negative
How often you give your child conscious attention?	7 (21%)	20 (59%)	7 (21%)	0 (0%)
Knowing how to take better care of yourself?	14 (41%)	19 (56%)	1 (3%)	0 (0%)
Actually taking better care of yourself?	8 (24%)	18 (53%)	8 (24%)	0 (0%)
Awareness of what is stressful in your life?	15 (44%)	13 (38%)	6 (18%)	0 (0%)
Awareness of stressful parenting situations at the time they are happening?	15 (44%)	14 (41%)	5 (15%)	0 (0%)
The frequency of parental stress?	15 (44%)	13 (38%)	6 (18%)	0 (0%)
The intensity of parenting stress or frustration?	16 (47%)	14 (41%)	4 (12%)	0 (0%)
Dealing with emotions while taking care of or parenting your child?	11 (32%)	19 (56%)	4 (12%)	0 (0%)
The ability to handle stressful parenting situations appropriately?	14 (41%)	18 (53%)	2 (6%)	0 (0%)
Being content with the relationship with your child?	15 (44%)	15 (44%)	4 (12%)	0 (0%)
The confidence you have in yourself as a mother?	14 (41%)	14 (41%)	6 (18%)	0 (0%)
Feeling hopeful as a mother?	14 (41%)	16 (47%)	4 (12%)	0 (0%)
	Likert scale ranging from 1 (not important) to 10 (enormously important)			
How important has the training been for you?	8.1 (1.6)			

Data are presented as *n* (%) or mean (standard deviation)

### Direct and Delayed Effects

Scores on all outcome measures at pretest, posttest, 8-week follow-up, and 1-year follow-up are displayed in Table 2. Results of multilevel models of treatment outcome predicted by measurement occasion are displayed in Table 3. Infant age and group were included as control variables if the effects of these variables were significant. As expected, mothers’ mindfulness (FFMQ), mindful parenting (IM-P), and self-compassion (SCS-3) were improved during Mindful with your baby (medium and large effects). Effects were stable in the 8-week follow-up period. At 1-year follow-up, only mindfulness and self-compassion improved further (large effects compared to pretest).

**Table 2:** Means and standard deviations of all dependent measures at all measurement occasions (the Mindful with your baby training took place between pretest and posttest)

Outcome variable	Pretest		Posttest		2-month follow-up		1-year follow-up	
	*n* = 37	*M* (*SD*)	*n* = 37	*M* (*SD*)	*n* = 37	*M* (*SD*)	*N* = 28	*M* (*SD*)
Mindfulness (FFMQ-SF)	36	2.9 (.8)	34	3.3 (.8)	31	3.4 (.7)	17	3.8 (.04)
Observing	36	3.4 (.9)	34	3.6 (.9)	31	3.7 (.9)	17	4.0 (.9)
Describing	36	3.5 (.8)	34	3.6 (.9)	31	3.7 (.9)	17	4.1 (.7)
Awareness	36	2.7 (.7)	34	3.2 (.8)	31	3.4 (.7)	17	3.9 (.6)
Non-judging of inner experience	36	2.6 (.7)	34	3.1 (1.0)	31	3.3 (.9)	17	3.6 (.8)
Non-reactivity	36	2.5 (.9)	34	3.0 (.7)	31	3.1 (.9)	17	3.3 (.7)
Mindful parenting (IM-P)	36	3.4 (.6)	34	3.6 (.5)	31	3.7 (.5)	18	3.8 (.5)
Listening with full attention	36	3.3 (.8)	34	3.6 (.7)	31	3.7 (.7)	18	3.5(.8)
Compassion for the child	36	4.4 (.6)	34	4.5 (.7)	31	4.4 (.8)	18	4.6 (.5)
Non-judgmental acceptance of parental functioning	36	2.5 (.8)	34	2.9 (.7)	31	3.1 (.7)	18	3.3 (.6)
Emotional non-reactivity in parenting	36	3.7 (.8)	34	3.8 (.9)	31	3.8 (.7)	18	4.1 (.8)
Emotional awareness of the child	36	3.5 (.9)	34	3.8 (.6)	31	4.0 (.6)	18	4.2 (.8)
Emotional awareness of the self	36	3.5 (.8)	34	3.7 (.8)	31	3.9 (.7)	18	4.0 (.8)
Self-compassion (SCS-3)	36	2.9 (1.0)	34	4.1 (1.4)	31	4.2 (1.2)	18	4.6 (1.2)
Well-being (WHO-5)	36	43.3 (19.6)	34	50.5 (24.4)	31	51.8 (22.4)	17	54.1 (13.4)
Psychopathology (ASR)	36	62.3 (10.4)	36	58.0 (10.9)	26	56.5 (11.8)	18	48.9 (7.9)
Internalizing psychopathology	36	66.7 (11.4)	36	61.3 (12.9)	26	60.5 (13.1)	18	53.7 (8.7)
Externalizing psychopathology	36	58.9 (9.1)	36	55.4 (8.9)	26	53.5 (11.6)	18	45.4 (6.9)
Withdrawn	36	59.9 (9.5)	36	57.4 (9.4)	26	58.5 (7.8)	18	53.9 (4.9)
Somatic complaints	36	62.5 (8.8)	36	59.0 (8.4)	26	59.0 (8.4)	18	55.5 (9.0)
Anxiety/depression	36	68.4 (9.6)	36	64.0 (11.6)	26	62.3 (12.3)	18	56.9 (6.8)
Rule-breaking behavior	36	56.5 (8.3)	36	55.8 (7.1)	26	56.2 (9.1)	18	52.0 (4.9)
Aggressive behavior	36	62.5 (6.3)	36	59.6 (6.4)	26	58.4 (6.8)	18	53.6 (3.9)
Intrusive	36	53.5 (5.2)	36	52.7 (4.8)	26	51.8 (4.4)	18	50.2 (.4)
Thought problems	36	60.4 (7.9)	36	57.8 (7.8)	26	56.7 (7.6)	18	51.7 (1.8)
Attention problems	36	66.4 (11.7)	36	63.0 (9.6)	26	62.5 (8.9)	18	58.2 (7.7)
Parenting stress and lack of confidence (PSI)								
Parenting stress	36	2.8 (1.0)	35	2.5 (.8)	26	2.4 (.8)	18	2.3 (.7)
Lack of confidence	36	2.9 (1.1)	35	2.5 (.9)	26	2.3 (.8)	18	2.0 (.6)
Parenting behavior (CPBQ)								
Warmth	36	4.2 (.6)	34	4.4 (.5)	31	4.4 (.6)	17	4.7 (.3)
Attention	36	4.2 (.8)	34	4.3 (.7)	31	4.2 (.8)	17	4.5 (.6)
Affection	36	4.6 (.6)	34	4.7 (.5)	31	4.6 (.7)	17	4.9 (.2)
Responsivity	36	3.8 (.6)	34	4.2 (.6)	31	4.1 (.6)	17	4.4 (.6)
Negativity	36	2.1 (.7)	34	2.0 (.6)	31	2.0 (.6)	17	1.8 (.6)
Rejection	36	1.8 (.7)	34	1.7 (.7)	31	1.8 (.7)	17	1.6 (.6)
Hostility	36	2.4 (.8)	34	2.1 (.7)	31	2.1 (.7)	17	2.0 (.7)
Infant temperament (IBQ-R VSF)								
Positive affectivity/surgency	32	4.9 (1.0)	33	5.3 (.8)	28	5.3 (.7)	–	–
Orienting/regulatory capacity	32	5.4 (.7)	33	5.6 (.8)	28	5.4 (.8)	–	–
Negative emotionality	32	4.1 (1.1)	33	4.3 (1.2)	28	4.3 (1.1)	–	–

Data are presented as mean (standard deviation). The ASR is *T* scores, the WHO-5 is percentage scores, and other scales are mean item scores (scale ranges were 1–5 for the FFMQ, IM-P, SCS-3, and CPBQ; 1–6 for the PSI; and 1–7 for the IBQ-R)

**Table 3:** Parameter estimates (and standard errors) and *F* values of multilevel models of treatment outcome predicted by measurement occasion (deviations from pretest) and control variables (infant age and training group)

	Intercept		Posttest		8-week follow-up		1-year follow-up		Infant age		Group
	*Β* (*SE*)	*F*	*Β* (*SE*)	*F*	*Β* (*SE*)	*F*	*Β* (*SE*)	*F*	*Β* (*SE*)	*F*	*F*
Mindfulness (FFMQ-SF)	−1.09 (.27)	28.44*	.56 (.12)	20.51*	.64 (.14)	19.97*	.95 (.16)	35.31*	–	–	4.85*
Observing	−.22 (.35)	1.91	.13 (.12)	1.12	.01 (.11)	.02	.34 (.15)	2.31*	–	–	3.28*
Describing	−.26 (.29)	6.71*	.21 (.12)	2.91^†^	.19 (.14)	1.72	.41 (.14)	8.51*	–	–	5.72*
Awareness	−.64 (.14)	19.76*	.66 (.13)	24.43*	.84 (.14)	37.58*	1.31 (.24)	42.63*	–	–	–
Non-judging of inner experience	−.96 (.32)	20.83*	.54 (.18)	9.07*	.72 (.17)	17.27*	.78 (.21)	13.26*	–	–	9.34*
Non-reactivity	−1.29 (.30)	15.09*	.53 (.14)	13.63*	.65 (.14)	2.07*	.57 (.21)	7.56*	–	–	3.87*
Mindful parenting (IM-P)	−1.49 (.33)	8.70*	.57 (.12)	20.52*	.69 (.14)	24.71*	.55 (.18)	9.74*	.24 (.11)	.11*	3.85*
Listening with full attention	−.32 (.17)	3.54^†^	.36 (.11)	9.97*	.53 (.13)	16.04*	.42 (.20)	4.48*	–	–	–
Compassion for the child	−.25 (.35)	.15	.29 (.14)	4.23*	−.01 (.20)	.00	.22 (.21)	1.12	.25 (.10)	6.24*	4.04*
Non-judgmental acceptance of parental functioning	−1.41 (.36)	10.82*	.58 (.18)	10.66*	.81 (.17)	23.34*	.72 (.21)	12.13*	–	–	2.39*
Emotional non-reactivity	−.76 (.39)	1.14	.19 (.16)	1.44	.21 (.14)	2.10	.28 (.22)	1.75	−.31 (.08)	15.12*	4.91*
Emotional awareness of child	−.45 (.20)	5.24*	.51 (.17)	8.72*	.66 (.18)	13.56*	.83 (.21)	15.18*	–	–	–
Emotional awareness of self	−.67 (.31)	3.09^†^	.31 (.18)	2.95^†^	.44 (.18)	5.83*	.19 (.27)	.49	–	–	3.58*
Self-compassion (SCS-3)	−.66 (.13)	26.77*	.82 (.12)	43.53*	.81 (.13)	39.08*	1.10 (.19)	32.94*	–	–	–
Well-being (WHO-5)	−1.48 (.31)	4.41*	.34 (.17)	4.23*	.40 (.14)	7.97*	.46 (.14)	11.03*	–	–	11.22*
Psychopathology (ASR)	.45 (.16)	7.91*	−.46 (.13)	11.93*	−.53 (.17)	10.25*	−.98 (.15)	42.40*	–	–	–
Internalizing	.42 (.15)	7.63*	−.45 (.16)	8.06*	−.47 (.16)	8.66*	−.89 (.16)	31.03*	–	–	–
Externalizing	.46 (.17)	7.42*	−.43 (.12)	13.23*	−.53 (.19)	7.77*	−1.12 (.16)	49.27*	–	–	–
Withdrawn	.29 (.42)	2.76	−.27 (.15)	3.14	−.06 (.17)	.14	−.47 (.17)	7.18*	–	–	4.48*
Somatic complaints	.75 (.37)	5.74*	−.38 (.16)	5.88*	−.30 (.17)	3.15	−.57 (.22)	6.76*	–	–	4.85*
Anxious/depressed	.72 (.36)	16.12*	−.49 (.13)	14.02*	−.65 (.15)	17.51*	−.77 (.16)	23.38*	–	–	3.51*
Rule-breaking behavior	.17 (.18)	.86	−.10 (.11)	.81	−.15 (.21)	.48	−.54 (.19)	8.16*	–	–	–
Aggressive behavior	.94 (.45)	8.92*	−.56 (.13)	17.82*	−.69 (.17)	15.80*	−1.31 (.17)	53.97*	–	–	3.52*
Intrusive	.27 (.18)	2.12	−.14 (.17)	.67	−.28 (.18)	2.39	−.77 (.17)	22.14*	–	–	–
Thought problems	.25 (.39)	6.95*	−.34 (.18)	3.62^†^	−.36 (.19)	−3.53†	−.75 (.19)	16.22*	–	–	3.00*
Attention problems	.31 (.18)	3.00^†^	−.30 (.16)	3.63^†^	−.44 (.15)	8.65*	−.68 (.18)	13.70*	–	–	–
Parenting stress and lack of confidence (PSI)											
Parenting stress	1.11 (.38)	1.65	−.25 (.15)	2.81	−.44 (.16)	4.48*	−.53 (.17)	9.49*	–	–	2.53*
Lack of confidence	1.73 (.35)	5.82*	−.37 (.17)	4.54*	−.59 (.17)	12.44*	−.75 (.15)	24.27*	–	–	3.76*
Parenting behavior (CPBQ)											
Warmth^a^	−.94 (.32)	3.07	.39 (.14)	7.77	.26 (.16)	2.52	.55 (.18)	9.06^†^	–	–	–
Attention	−1.24 (.38)	.79	.19 (.11)	2.92^†^	.08 (.15)	.29	.22 (.17)	1.64	.32 (.11)	7.72*	6.25*
Affection	−.49 (.44)	.09	.18 (.13)	2.00	.11 (.19)	.31	.47 (.14)	11.23*	.32 (.10)	10.30*	3.25*
Responsivity	−.45 (.15)	8.86*	.68 (.13)	27.23*	.48 (.15)	9.91*	.86 (.19)	21.57*	–	–	–
Negativity	.70 (.36)	2.67	−.28 (.14)	3.98^†^	−.21 (.19)	1.28	−.42 (.19)	5.01*	–	–	3.93*
Rejection	.45 (.30)	.06	−.07 (.15)	.19	.06 (.21)	.10	−.11 (.15)	.56	–	–	5.13*
Hostility	.64 (.39)	3.64	−.39 (.14)	3.64*	−.41 (.18)	5.16*	−.48 (.20)	5.52*	–	–	3.14*
Infant temperament (IBQ-R VSF)											
Positive affectivity/surgency	−.33 (.18)	3.52^†^	.48 (.15)	10.00*	.51 (.15)	10.72*	–	–	.34 (.12)	8.23*	–
Orienting/regulatory capacity	−.14 (.15)	.86	.35 (.19)	3.30^†^	.06 (.18)	.11	–	–	.38 (.11)	11.55*	–
Negative emotionality	−.69 (.34)	3.13^†^	.25 (.16)	2.33	.19 (.12)	2.49	–	–	–	–	2.97*

*B* = parameter estimate, which can be interpreted as Cohen’s *d* effect size of change. Control variables infant age and group were only retained in the models when significant (fixed effects)

^†^ *p* < .10; *p < .05

^a^The random effect for group was significant (for group 2) in this model predicting maternal warmth (*B* (*SE*) = 1.21 (.57), 95% CI [.49, 3.04], *p* = .032), which means that there was significant random variance in the effect of the training on their warm behavior towards their infant in group 2. This effect was therefore included in the model as a potential confound

An improvement was seen in maternal well-being (WHO-5; small effect) and maternal psychopathology (ASR, small effect at posttest, medium effect at 8-week follow-up, and large effect at 1-year follow-up). Improvement in maternal parenting stress (PSI) was significant only at the 8-week follow-up (small effect) and 1-year follow-up (medium effect), while improvement in parenting confidence (PSI) already started at posttest (small effect) and improved further over time (medium effect at both follow-up measurements). Of maternal warm and negative behavior towards the infant (CPBQ), two subscales showed improvement at posttest, namely responsivity and hostility (medium and small effects, respectively). Compared to pretest, these subscales also showed improvement at both follow-up measurement occasions (small to large effects). Subscale affection only improved at 1-year follow-up (small to medium effects), and the other CPBQ subscales (attention and rejection) did not improve.

Improvement in infant temperamental behavior was also reported. At both posttest and 8-week follow-up, infant positive affectivity/surgency increased (medium effect), whereas on the other components orienting/regulatory capacity and negative emotionality, no significant improvement occurred.

Of the 37 research participants, 24 (65%) received any other form of psychological intervention during the training or in the 8-week follow-up period. When analyses that showed significant effects of measurement occasion at posttest or follow-up on main outcomes in the full group (mindfulness, mindful parenting, self-compassion, well-being, psychopathology, parenting stress, parenting lack of confidence, warm behavior, and infant positive affectivity/surgency) were repeated for the subgroup of mothers that did not receive any other psychological intervention, effect sizes of outcomes were similar, except for maternal well-being (WHO-5), mindfulness (FFMQ), and IBQ component positive affectivity/surgency, showing a larger effect size than in the full group, and psychopathology (ASR), showing a smaller effect size than in the full group.

## Discussion

In this study, we aimed to evaluate Mindful with your baby, a mindful parenting training for mothers with infants aged 0 to 18 months. We hypothesized that Mindful with your baby would be acceptable for the participants and would improve maternal mindfulness, mindful parenting, self-compassion, well-being, psychopathology, parenting stress, lack of confidence, warmth and negativity towards the baby, and infant temperament and that these effects would maintain for 8 weeks and 1 year after the training had ended.

With respect to the first hypothesis, it can be concluded that Mindful with your baby is a feasible and acceptable program for mothers with infants, who experience stress in motherhood. Dropout and attendance rates were acceptable, all mothers who completed the evaluation form felt that they had gotten something of lasting value from the training, and participants rated the importance of the training with an average of 8.1 (scale 1–10).

In line with our second hypothesis, mothers became more mindful, both in general and in their parenting, and more compassionate towards themselves during the training, and this improvement was maintained during the 8-week follow-up period (medium to large effect sizes). Effects on mindfulness and mindful parenting are smaller than those on the regular mindful parenting training (Meppelink et al. 2016). It is important to note that the results were obtained when meditating with the babies present during most sessions, which gives a very different atmosphere than in a group of only parents, as was the case in Meppelink et al.’s study. Also, the meditations that were practiced at home were shorter (about 15 min) than in the regular mindful parenting training (about 30 min). Mindfulness and self-compassion (but not mindful parenting) further improved during the 1-year follow-up period (large effect sizes). Due to the limited number of participants that filled in the 1-year follow-up (about 60% of the research participants that had already been administered a 1-year follow-up measurement), results of the 1-year follow-up should be interpreted carefully.

There was a significant improvement in maternal well-being (small effect size). At pretest, the mean score was below the cutoff score for low mood and thus indicative of reduced well-being. At posttest and follow-up, mean scores were above this cutoff score. Also, there were improvements in maternal psychopathology. At posttest, somatic complaints, anxious/depressed, and aggressive behavior improved (small to medium effect sizes). At 1-year follow-up, all aspects of psychopathology had improved (medium to very large effects). Mean maternal psychopathology scores at pretest were at a clinical level for the internalizing scale and at a subclinical level for the total scale, the anxious/depressed subscale, and attention subscale, whereas at posttest and both follow-ups, all mean (sub)scale scores were in the normal range. It is not possible to rule out the possibility of spontaneous recovery of the mothers, related to the passage of time after giving birth and adjustment to motherhood. However, infant age was not related to any of the psychopathology outcomes, suggesting that passage of time alone was not responsible for recovery. Also, the improvement of maternal mindfulness during the 1-year follow-up period, and earlier research showing that mindfulness is the mechanism of action for psychological outcomes of mindfulness-based interventions (Gu et al. 2015; Meppelink et al. 2016), suggest that the further improvement in psychopathology up to 1 year after the training may be (partly) attributable to Mindful with your baby.

Maternal confidence in parenting improved over time (from a small to medium effect size), but maternal parenting stress took some more time to improve (small and medium effect sizes at 8-week and 1-year follow-up, respectively). Maternal responsivity improved at all three measurement occasions (small to large effects), maternal affection only at 1-year follow-up (small effect), and the last subscale that maternal warmth comprised of, attention, did not improve. Neither did maternal rejection, but the other subscale that maternal negative behavior was comprised of hostility did show improvement at the three time points (small effects). Mothers seem to recognize not only a change in internal experience but also a translation of this change to the way they behave towards their baby. It would be of interest to study whether this increased responsivity and decreased hostility can be observed in mother-child interactions before and after Mindful with your baby.

In this study, infant behavior was measured with a questionnaire measuring temperament. This choice might raise concerns, as most dimensions of temperament are regarded as relatively stable (Rothbart et al. 2000). However, it has been recognized that parental factors may influence infant temperament (Pesonen et al. 2005; Tikotzky et al. 2010). Rothbart (1991) distinguished two components of temperament, self-regulation, and reactivity. In mindfulness-based interventions, both self-regulation and non-reactivity are trained. Possibly, these qualities in mothers may support the development of the same qualities in infants. Yu and Smith (2016) showed that the joint attention between mothers and 1-year-old infants that was sustained by the mothers extended the duration of the attention of the infants. This is an example of a self-regulatory ability of the infant that develops in relationship with the mother.

Earlier research found an association between maternal mindfulness in pregnancy and infant temperament (Van den Heuvel et al. 2015a, b). The current study shows that also after birth, the development of temperament, namely the development of positive affectivity, may be influenced positively when mothers practice mindfulness and mindful parenting. Although infant age was a significant covariate, the effect size of measurement occasion was in the medium range both at posttest and follow-up. A possible explanation is that babies become more positive when their mothers are more attentive towards them. Another explanation of this change is that, because mothers become more able to focus their attention on their infant with openness and curiosity, they might be able to recognize positive affect in the infant better. This might open opportunities for mother and infant synchrony, dyadic interactions that are mutually regulated, harmonious, and reciprocal (Reyna and Pickler 2009).

Infant temperament was reported by the participating mother. The question therefore can be asked, whether infant temperamental behavior really changed or whether mothers’ perception changed. Possibly, perception might be negatively biased before the training, due to psychopathology of the mother (Najman et al. 2000), or positively biased after the training. Where a bias to exist, this would not necessarily make the findings on infant temperament less important, as parental perceptions shape parental behaviors (Pauli-Pott et al. 2003; Tikotzky and Sadeh 2009) and thus the relationship with the child, which may lead to actual changes in infant temperament.

Infant age was not only predictive of infant behavior (positive affectivity/surgency and orienting/regulatory capacity) but was also a significant covariate in several models predicting maternal behavior (attention and affection) and mindful parenting. Mothers with an older infant gave their infants more attention, showed more affection, were more mindful in their parenting, and showed more compassion for the child. They were, however, more emotionally reactive. Possibly, emotional reactivity starts to play a bigger role when babies slowly develop into toddlers and start to show more challenging behavior.

Group was also used as a control variable; the fixed effect was significant in the majority of the models. The group that participants are part of seems to matter in the effect of the training. This difference in effect of the training between groups may depend not only on differences in trainers and IMH specialists but also on the composition of the groups, openness of the participants, and group processes.

### Limitations

The findings of the current study should be interpreted, considering the following limitations. First, the lack of a control group or waitlist condition limits our conclusions on the beneficial effects of the training. People who suffer from stress or psychopathology tend to improve in functioning over time, especially when they decide to seek help. Second, the effects that were shown in this paper may be (partly) attributable to other factors, such as adjustment to motherhood with time or developmental stage at which the infant is in. Although infant age was shown to not be a significant covariate in this study, another study design addressing the additional dimensions of overall adjustment to parenthood is needed to rule out this possibility. The fact that the group of women with babies that participated in Mindful with your baby was not a randomly chosen group of mothers with elevated stress or mental health problems, babies with (regulation) problems, or mother-infant interaction problems is also a limitation. The participating mothers were not only from a sample referred for treatment but also actively chose for this training. Therefore, they may have been more motivated to benefit than referred mother who did not choose for this training. Another limitation is that a substantial proportion of participants (61% of the training participants and 65% of the research participants) received other forms of psychological help during the training and/or in the follow-up period. Because the Mindful with your baby program was new, and the effectiveness unclear, we found it unethical to withhold possible additional support from vulnerable participants in this essential time of their life. As a result of this practical decision, it remains unclear how much of the reported change is a result of mindfulness intervention. To reach firm conclusions about the effectiveness of this intervention, future studies should consider including a control group or waitlist condition in to the design. Yet, another limitation is the fact that all measurements were done only by the mothers who participated in the training. Given that they spend time and effort to the training, they may have been biased to attribute positive effects. Also, the use of questionnaires has its limitations. Parent report of child behavior may be more biased for parents of infants than parents of older children, because of lack of knowledge about normal development in infancy, and hesitance to report problem behavior in infants (Carter et al. 2009). Also, to reliably measure parent-child interaction, the use of questionnaires is not sufficient (Miron et al. 2009). Future studies should include observational measures (for example, sensitivity observations) and multiple informants (for example, the father reporting on child functioning and on maternal functioning).

Nevertheless, the current study provides initial evidence supporting the idea that Mindful with your baby is a promising intervention for mothers with infants who experience stress in motherhood or mental health problems. The attendance rates and positive evaluations suggest that Mindful with your baby is a feasible and acceptable intervention. Furthermore, the training seems to be effective, as was shown by improvements in maternal mindfulness, mindful parenting, self-compassion, psychopathology, well-being, parenting stress, lack of confidence, warmth, responsivity, and hostility. Infants also seem to benefit from the intervention, as was shown by improved positive affectivity.

## References

[CR1] Achenbach TM, Rescorla LA (2003). Manual for the ASEBA adult forms & profiles.

[CR2] Abidin RR (1983). Parenting Stress Index manual.

[CR3] Alcorn KL, O’Donovan A, Patrick JC, Creedy D, Devilly GJ (2010). A prospective longitudinal study of the prevalence of post-traumatic stress disorder resulting from childbirth events. Psychological Medicine.

[CR4] Baer RA, Smith GT, Hopkins J, Krietemeyer J, Toney L (2006). Using self-report assessment methods to explore facets of mindfulness. Assessment.

[CR5] Bagiella E, Sloan RP, Heitjan DF (2000). Mixed-effects models in psychophysiology. Psychophysiology.

[CR6] Bakermans-Kranenburg MJ, Van IJzendoorn MH, Juffer F (2003). Less is more: meta-analyses of sensitivity and attachment interventions in early childhood. Psychological Bulletin.

[CR7] Bennett SS, Indman P (2003). Beyond the blues: a guide to understanding and treating prenatal and postpartum depression.

[CR8] Bögels SM, Hellemans J, van Deursen S, Römer M, van der Meulen R (2014). Mindful parenting in mental health care: effects on parental and child psychopathology, parental stress, parenting, coparenting, and marital functioning. Mindfulness.

[CR9] Bögels S, Restifo K (2013). Mindful parenting: a guide for mental health practitioners.

[CR10] Braeken MAKA, Jones A, Otte RA, Nyklíček I, Van den Bergh BRH (2016). Potential benefits of mindfulness during pregnancy on maternal autonomic nervous system function and infant development. Psychophysiology.

[CR11] Britton JR (2011). Infant temperament and maternal anxiety and depressed mood in the early postpartum period. Women & Health.

[CR12] de Brock AJLL, Vermulst AA, Gerris JRM, Abidin RR (1992). NOSI, Nijmeegse Ouderlijke Stress Index.

[CR13] Carter AS, Godoy L, Marakovitz SE, Briggs-Gowan MJ, Zeanah CH (2009). Parent reports and infant–toddler mental health assessment. Handbook of infant mental health.

[CR14] Cree M (2010). Compassion focused therapy with perinatal and mother-infant distress. International Journal of Cognitive Therapy.

[CR15] Crnic KA, Greenberg MT, Slough NM (1986). Early stress and social support influences on mothers’ and high-risk infants’ functioning in late infancy. Infant Mental Health Journal.

[CR16] De Bruin EI, Topper M, Muskens JG, Bögels SM, Kamphuis JH (2012). Psychometric properties of the Five Facets Mindfulness Questionnaire (FFMQ) in a meditating and a non-meditating sample. Assessment.

[CR17] De Bruin EI, Zijlstra BJ, Geurtzen N, van Zundert RM, van de Weijer-Bergsma E, Hartman EE (2014). Mindful parenting assessed further: psychometric properties of the Dutch version of the Interpersonal Mindfulness in Parenting Scale (IM-P). Mindfulness.

[CR18] De Vibe, M., Bjørndal, A., Tipton, E., Hammerstrøm, K. T., & Kowalski, K. (2012). Mindfulness based stress reduction (MBSR) for improving health, quality of life, and social functioning in adults. *Campbell Systematic Reviews, 8*(3).

[CR19] Dimidjian S, Goodman S (2009). Nonpharmacologic intervention and prevention strategies for depression during pregnancy and the postpartum. Clinical Obstetrics and Gynecology.

[CR20] Douglas PS, Hill PS (2013). Behavioral sleep interventions in the first six months of life do not improve outcomes for mothers or infants: a systematic review. Journal of Developmental & Behavioral Pediatrics.

[CR21] Duncan LG (2007). Assessment of mindful parenting among parents of early adolescents: development and validation of the interpersonal mindfulness in parenting scale.

[CR22] Duncan LG, Bardacke N (2010). Mindfulness-based childbirth and parenting education: promoting family mindfulness during the perinatal period. Journal of Child and Family Studies.

[CR23] Dunn C, Hanieh E, Roberts R, Powrie R (2012). Mindful pregnancy and childbirth: effects of a mindfulness-based intervention on women’s psychological distress and well-being in the perinatal period. Archives of Women’s Mental Health.

[CR24] Feeney JA (2003). Adult attachment, involvement in infant care, and adjustment to new parenthood. Journal of Systemic Therapies.

[CR25] Gartstein MA, Rothbart MK (2003). Studying infant temperament via the revised Infant Behavior Questionnaire. Infant Behavior and Development.

[CR26] Gavin, N. I., Gaynes, B. N., Lohr, K. N., Meltzer-Brody, S., Gartlehner, G., & Swinson, T. (2005). Perinatal depression: a systematic review of prevalence and incidence. *Obstetrics & Gynecology*, *106*(5, Part 1), 1071–1083. 10.1097/01.AOG.0000183597.31630.db 16260528

[CR27] Gentile S, Rossi A, Bellantuono C (2007). SSRIs during breastfeeding: spotlight on milk-to-plasma ratio. Archives of Women’s Mental Health.

[CR28] Gu J, Strauss C, Bond R, Cavanagh K (2015). How do mindfulness-based cognitive therapy and mindfulness-based stress reduction improve mental health and wellbeing? A systematic review and meta-analysis of mediation studies. Clinical Psychology Review.

[CR29] Hajos TRS, Pouwer F, Skovlund SE, Den Oudsten BL, Geelhoed-Duijvestijn PHLM, Tack CJ, Snoek FJ (2013). Psychometric and screening properties of the WHO-5 Well-Being Index in adult outpatients with type 1 or type 2 diabetes mellitus. Diabetic Medicine.

[CR30] Hassan, G. (2014). Mindful beginnings: the benefits of mindfulness in early parenting. https://medium.com/@DrGinaHassan/mindful-beginnings-the-benefits-of-mindfulness-in-early-parenting-7f66e97c588f#.ptrztidq1. Accessed 30 January 2017.

[CR31] Henrichs J, Schenk JJ, Schmidt HG, Velders FP, Hofman A, Jaddoe VWV, Verhulst FC, Tiemeier H (2009). Maternal pre-and postnatal anxiety and infant temperament. The generation R study. Infant and Child Development.

[CR32] Kabat-Zinn J (1990). Full catastrophe living: the program of the stress reduction clinic at the University of Massachusetts Medical Center.

[CR33] Kabat-Zinn J (1994). Wherever you go, there you are.

[CR34] Kabat-Zinn M, Kabat-Zinn J (1997). Everyday blessings: the inner work of mindful parenting.

[CR35] Kersten-Alvarez LE, Hosman CM, Riksen-Walraven JM, Van Doesum K, Hoefnagels C (2011). Which preventive interventions effectively enhance depressed mothers’ sensitivity? A meta-analysis. Infant Mental Health Journal.

[CR36] Krongold KS (2011). Mindfulness-based prenatal care and postnatal mother-infant relationships (doctoral dissertation).

[CR37] Laurent HK, Duncan LG, Lightcap A, Khan F (2016). Mindful parenting predicts mothers’ and infants’ hypothalamic-pituitary-adrenal activity during a dyadic stressor. Developmental Psychology.

[CR38] Lazarus RS, Folkman S, Appley MH, Trumbull RA (1986). Cognitive theories of stress and the issue of circularity. Dynamics of stress.

[CR39] Luhmann M, Hofmann W, Eid M, Lucas RE (2012). Subjective well-being and adaptation to life events: a meta-analysis. Journal of Personality and Social Psychology.

[CR40] Lupien SJ, McEwen BS, Gunnar MR, Heim C (2009). Effects of stress throughout the lifespan on the brain, behaviour and cognition. Nature Reviews Neuroscience.

[CR41] Majdandžić M, Vente W, Bögels SM (2015). Challenging parenting behavior from infancy to toddlerhood: etiology, measurement, and differences between fathers and mothers. Infancy.

[CR42] Meppelink R, de Bruin EI, Wanders-Mulder FH, Vennik CJ, Bögels SM (2016). Mindful parenting training in child psychiatric settings: heightened parental mindfulness reduces parents’ and children’s psychopathology. Mindfulness.

[CR43] Miron D, Lewis ML, Zeanah CH, Zeanah CH (2009). Clinical use of observational procedures in early childhood relationship assessment. Handbook of infant mental health.

[CR44] Misri S, Reebye P, Corral M, Milis L (2004). The use of paroxetine and cognitive-behavioral therapy in postpartum depression and anxiety: a randomized controlled trial. The Journal of Clinical Psychiatry.

[CR45] Nagata M, Nagai S, Sobajima H, Ando T, Nishide Y, Honjo S (2000). Maternity blues and attachment to children in mothers of full-term normal infants. Acta Psychiatrica Scandinavica.

[CR46] Najman JM, Williams GM, Nikles J, Spence S, Bor W, O’Callaghan M, Le Brocque R, Andersen MJ (2000). Mothers’ mental illness and child behavior problems: cause-effect association or observation bias?. Journal of the American Academy of Child & Adolescent Psychiatry.

[CR47] Neff KD (2003). The development and validation of a scale to measure self-compassion. Self and Identity.

[CR48] Nelson AM (2003). Transition to motherhood. Journal of Obstetric, Gynecologic, & Neonatal Nursing.

[CR49] Pesonen AK, Räikkönen K, Strandberg TE, Järvenpää AL (2005). Continuity of maternal stress from the pre-to the postnatal period: associations with infant’s positive, negative and overall temperamental reactivity. Infant Behavior and Development.

[CR50] Pauli-Pott U, Mertesacker B, Bade U, Haverkock A, Beckmann D (2003). Parental perceptions and infant temperament development. Infant Behavior and Development.

[CR51] Putnam SP, Gartstein MA, Rothbart MK (2006). Measurement of fine-grained aspects of toddler temperament: the early childhood behavior questionnaire. Infant Behavior and Development.

[CR52] Putnam SP, Helbig AL, Gartstein MA, Rothbart MK, Leerkes E (2014). Development and assessment of short and very short forms of the Infant Behavior Questionnaire—revised. Journal of Personality Assessment.

[CR53] Raes F, Pommier E, Neff KD, Van Gucht D (2011). Construction and factorial validation of a short form of the Self-Compassion Scale. Clinical Psychology & Psychotherapy.

[CR54] Reck C, Stehle E, Reinig K, Mundt C (2009). Maternity blues as a predictor of DSM-IV depression and anxiety disorders in the first three months postpartum. Journal of Affective Disorders.

[CR55] Reyna BA, Pickler RH (2009). Mother-infant synchrony. Journal of Obstetric, Gynecologic, & Neonatal Nursing.

[CR56] Reynolds D (2003). Mindful parenting: a group approach to enhancing reflective capacity in parents and infants. Journal of Child Psychotherapy.

[CR57] Ross LE, McLean LM, Psych C (2006). Anxiety disorders during pregnancy and the postpartum period: a systematic review. Depression.

[CR58] Rothbart MK, Strelau J, Angleitner A (1991). Temperament: a developmental framework. Explorations in temperament: international perspectives on theory and measurement.

[CR59] Rothbart MK, Derryberry D, Hershey K, Molfese VJ, Molfese DL (2000). Stability of temperament in childhood: laboratory infant assessment to parent report at seven years. Temperament and personality development across the life span.

[CR60] Segal ZV, Williams JMG, Teasdale JD (2002). Mindfulness-based cognitive therapy for depression: a new approach to relapse prevention.

[CR61] Segal ZV, Williams JMG, Teasdale JD (2012). Mindfulness-based cognitive therapy for depression.

[CR62] Shaddix C (2014). *An interpretative phenomenological study of the experience of parents who attended a mindfulness-based childbirth and parenting program* (doctoral dissertation).

[CR63] Sharma V, Sommerdyk C (2013). Are antidepressants effective in the treatment of postpartum depression? A systematic review. Prim. Care Companion CNS Disord.

[CR64] Siegel DJ (2007). The mindful brain: reflection and attunement in the cultivation of well-being.

[CR65] Siegel DJ, Hartzell M (2003). Parenting from the inside out: how a deeper self-understanding can help you raise children who thrive.

[CR66] Tikotzky L, Chambers AS, Gaylor E, Manber R (2010). Maternal sleep and depressive symptoms: links with infant negative affectivity. Infant Behavior and Development.

[CR67] Tikotzky L, Sadeh A (2009). Maternal sleep-related cognitions and infant sleep: a longitudinal study from pregnancy through the 1st year. Child Development.

[CR68] Topp CW, Østergaard SD, Søndergaard S, Bech P (2015). The WHO-5 Well-Being Index: a systematic review of the literature. Psychotherapy and Psychosomatics.

[CR69] Van den Heuvel MI, Johannes MA, Henrichs J, Van den Bergh BRH (2015). Maternal mindfulness during pregnancy and infant socio-emotional development and temperament: the mediating role of maternal anxiety. Early Human Development.

[CR70] Van den Heuvel MI, Donkers FC, Winkler I, Otte RA, Van den Bergh BR (2015). Maternal mindfulness and anxiety during pregnancy affect infants’ neural responses to sounds. Social Cognitive and Affective Neuroscience.

[CR71] Vieten C, Astin J (2008). Effects of a mindfulness-based intervention during pregnancy on prenatal stress and mood: results of a pilot study. Archives of Women’s Mental Health.

[CR72] Yu C, Smith LB (2016). The social origins of sustained attention in one-year-old human infants. Current Biology.

